# Evaluation of *core* Biomarkers of Alzheimer’s disease in saliva and plasma measured by chemiluminescent enzyme immunoassays on a fully automated platform

**DOI:** 10.1038/s41598-024-66923-z

**Published:** 2024-07-12

**Authors:** Luisa Agnello, Rosaria Vincenza Giglio, Fabio Del Ben, Tommaso Piccoli, Tiziana Colletti, Concetta Scazzone, Bruna Lo Sasso, Anna Maria Ciaccio, Caterina Maria Gambino, Giuseppe Salemi, Marcello Ciaccio

**Affiliations:** 1 Institute of Clinical Biochemistry, Clinical Molecular Medicine, and Clinical Laboratory Medicine, Department of Biomedicine, Neurosciences, and Advanced Diagnostics (BIND), University of Palermo, Palermo, Italy; 2 Department of Laboratory Medicine, University Hospital “P. Giaccone”, Palermo, Italy; 3 Immunopathology and Cancer Biomarkers, Centro di Riferimento Oncologico di Aviano (CRO)-IRCCS, Aviano, Italy; 4 Neurology Unit, Department of Biomedicine, Neuroscience, and Advanced Diagnostics (BIND), University of Palermo, Palermo, Italy; 5 Department of Biological, Chemical, and Pharmaceutical Sciences and Technologies (STEBICEF), University of Palermo, Palermo, Italy; 6 Internal Medicine and Medical Specialties “G. D’Alessandro”, Department of Health Promotion, Maternal, and Infant Care, University of Palermo, Palermo, Italy

**Keywords:** Beta-amyloid, Tau, AD, Biomarker, Plasma, Saliva, Fujirebio, Molecular biology, Biomarkers, Diseases, Neurology

## Abstract

Cerebrospinal fluid (CSF) core biomarkers of Alzheimer’s disease (AD), including amyloid peptide beta-42 (Aβ42), Aβ42/40 ratio, and phosphorylated tau (pTau), are precious tools for supporting AD diagnosis. However, their use in clinical practice is limited due to the invasiveness of CSF collection. Thus, there is intensive research to find alternative, noninvasive, and widely accessible biological matrices to measure AD *core* biomarkers. In this study, we measured AD *core* biomarkers in saliva and plasma by a fully automated platform. We enrolled all consecutive patients with cognitive decline. For each patient, we measured Aβ42, Aβ40, and pTau levels in CSF, saliva, and plasma by Lumipulse G1200 (Fujirebio). We included forty-two patients, of whom 27 had AD. Levels of all biomarkers significantly differed in the three biofluids, with saliva having the lowest and CSF the highest levels of Aβ42, Aβ40, and pTau. A positive correlation of pTau, Aβ42/40 ratio, and pTau/Aβ42 ratio levels in CSF and plasma was detected, while no correlation between any biomarker in CSF and saliva was found. Our findings suggest that plasma but not saliva could represent a surrogate biofluid for measuring *core* AD biomarkers. Specifically, plasma Aβ42/40 ratio, pTau/Aβ42 ratio, and pTau could serve as surrogates of the corresponding CSF biomarkers.

## Introduction

Alzheimer’s disease (AD) is the most common cause of dementia, accounting for 60–70% of cases, and a leading cause of disability worldwide^1^. Due to the increase in population growth and ageing, the number of individuals affected by dementia is expected to spread in 2050, exceeding 152 million cases^1^. According to the World Health Organization and Alzheimer Disease International Report, AD is regarded as a “global public health priority”^2^.

The pathological hallmarks of AD are extracellular β-Amyloid senile plaques, consisting of amyloid peptide beta-42 (Aβ42) deposition, and intracellular neurofibrillary tangles consisting of tau hyperphosphorylated (pTau)^3,4^. A definite diagnosis of AD relies on detecting its underlying pathologic processes, which can be documented *post-mortem* by brain examination or in vivo by biomarkers (imaging and molecular)^5^. Thus, biomarkers represent a precious tool for AD diagnosis.

In the field of molecular biomarkers, cerebrospinal fluid (CSF) Aβ42, Aβ42/40 ratio, pTau, and total tau (tTau), named AD *core* biomarkers, represent the gold standard for AD diagnosis. Specifically, according to the National Institute on Aging and Alzheimer's Association (NIA-AA) criteria, the AD diagnosis can be made, independently from the clinical stage, by detecting low CSF Aβ42 and Aβ42/40 ratio and high CSF pTau and tTau levels^5^. However, the use of CSF biomarkers in clinical practice is hampered by several issues, especially the invasiveness and the need for specialized personnel for CSF collection^5–9^. Thus, intensive research is ongoing to find alternative, noninvasive, and widely accessible biological matrices to measure AD *core* biomarkers.

Saliva and plasma represent two ideal biofluids, being easy to obtain, noninvasive, and their collection do not require hospitalization.

Over the past decade, significant advances in measuring AD *core* biomarkers have been made with the development of sensitive technologies to quantify very low levels. Thus, several Authors evaluated the performance of AD *core* biomarkers in plasma using different technologies, achieving promising results^10,11^. However, only a few Authors have explored the potential of using saliva as an alternative biofluid, leading to inconsistent findings^12^. In 2010, Bermejo-Pareja et al. first measured Aβ42 levels in saliva^13^ and, a year later, Shi et al. showed the presence of tau in saliva^14^. Since then, some Authors have measured AD *core* biomarkers, achieving heterogeneous findings^12^. Such discrepancies are in part related to pre-analytical issues due to saliva collection, treatment, and storage; the analytical method, being enzyme-linked immunosorbent assay (ELISA) the most used. However, there are no standardized and validated ELISA protocols for saliva analysis^15^; different clinical diagnostic criteria of AD.

The fully automated platform Lumipulse G based on the chemiluminescent enzyme immunoassays (CLEIA) method, widely used to measure CSF AD *core* biomarkers in clinical practice worldwide, has recently developed assays to measure Aβ42, Aβ40, and pTau in plasma. Since these assays have high sensitivity, we measured Aβ40, Aβ42, and pTau levels in plasma and saliva of patients with cognitive decline. Additionally, we compared plasma and saliva biomarkers’ levels with the respective CSF levels to evaluate the possible correlation of biomarkers among the different biological matrices.

## Material and methods

### Study population

We performed a prospective observational study at the University Hospital “P. Giaccone”, Palermo, Italy. We considered eligible all consecutive patients with cognitive decline and a suspicion of AD attending the Unit of Neurology from January to December 2023. All samples were analyzed at the Institute of Clinical Biochemistry, Clinical Molecular Medicine, and Clinical Laboratory Medicine, Department of Biomedicine, Neurosciences, and Advanced Diagnostics, University of Palermo, Palermo, Italy.

The diagnosis of AD or other neurological diseases was made by an expert neurologist based on medical history, clinical examination, neuropsychological testing, neuroimaging, fluorodeoxyglucose positron emission tomography (PET), and CSF biomarkers findings, according to the recent guidelines^5,16,17^.

All clinical and biological assessments were carried out in accordance with the Declaration of Helsinki, and the study was approved by the Ethics Committee of the University Hospital of Palermo (Nr. 02/2023). All participants gave informed written consent.

### Sample collection

We collected three different biological matrices for each patient: CSF, plasma, and saliva.

The collection of all biological matrices was made between 8:00 a.m. and 10:00 a.m. in a fasted state. Additionally, all patients were asked to refrain from eating, drinking, smoking, or using oral hygiene before collection (at least for 8 h). We documented the consumption of alcohol, caffeine, nicotine, and medication in the previous 12 h.

CSF was obtained by a lumbar puncture at the L3/4 or L4/5 interspace using a 21-gauge needle. It was collected in polypropylene tubes, centrifuged at 500*g* for 20 min, aliquoted in polypropylene tubes, and stored at − 80 °C until analysis, according to international consensus protocols^18^.

Whole blood was collected through venipuncture immediately before saliva sampling in K_3_-EDTA tubes, centrifuged at 2.500*g* for 10 min. The obtained plasma was collected, aliquoted in polypropylene tubes, and stored at − 80 °C until analysis.

After checking the oral cavity to exclude the presence of wounds, lacerations, or inflammatory processes (periodontitis), patients were requested to rinse their mouth with water before providing unstimulated saliva by spitting into a 50 ml polypropylene falcon tube (we get about 3 ml). The collected samples were immediately placed on ice and centrifuged at 1.500*g* for 5 min. After centrifugation, samples were divided into two aliquots in polypropylene tubes; (i) untreated: the aliquot was immediately stored at − 80 °C and; (ii) treated: the aliquot was added with thioflavin S (0.5 mg, Sigma, St. Louis, MO, USA) to prevent Aβ42 aggregation, and sodium azide (0.5 mg, Fischer Scientific, Suwanee, GA, USA) to prevent bacterial growth, before storing at − 80 °C. Before analysis, after thawing, saliva samples were centrifuged at 1.500*g* for 5 min. and the supernatant was analyzed.

### Biochemical analysis

The Aβ42, Aβ40, and pTau levels in all biological matrices, i.e. CSF, saliva, and plasma, were measured by CLEIA using the fully automated platform Lumipulse (Lumipulse G1200 analyzer, Fujirebio Inc. Europe, Gent, Belgium), according to the manufacturer’s instructions.

CSF Aβ42, Aβ40, and tau phosphorylated at threonine 181 levels were analyzed as part of the clinical routine using the following kits: Lumipulse G β-Amyloid 1–40 CSF, Lumipulse G β-Amyloid 1–42 CSF, and Lumipulse G pTau 181 CSF, respectively. The limit of detection (LoD) was 6.7 pg/mL for Aβ40, 2.78 pg/mL for Aβ42, and 0.282 pg/mL for pTau. The total precision of the assays (%Coefficient Variation [CV]) was 2–3.9 for Aβ40, 2.6–4.5 for Aβ42, and 2.2–8.3 for pTau.

Plasma and saliva Aβ42, Aβ40, and pTau levels were analyzed using the following kits: Lumipulse G β-Amyloid 1–40 Plasma, Lumipulse G β-Amyloid 1–42 Plasma, and Lumipulse G pTau 181 Plasma, respectively. The LoD was ≤ 0.44 pg/mL for Aβ40, ≤ 0.37 pg/mL for Aβ42, and 0.052 pg/mL for pTau. The total precision of the assays (%CV) was 2.6–4.6 for Aβ40, 4–5.6 for Aβ42, and 2.3–3.9 for pTau in plasma, and 61–9 for Aβ40, 97–14 for Aβ42, and 62–18 for pTau in saliva.

### Statistical analysis

Statistical analysis and visualization were performed by R version 4.3.2 (2023-10-31) using the packages ggstatsplot 0.12.2, ggplot2 3.5.0, tidyverse 2.0.0, mcr 1.3.3. Normality distribution was assessed preliminarily by q-q plot, Shapiro–Wilk test, and Kolmogorov–Smirnov test. Most variables were not normally distributed. Given these results, we opted for nonparametric descriptive statistics and tests. Differences among groups were tested by Kruskal Wallis test, with pairwise comparison tests adjusted with Holm method. Correlation matrix displayed Spearman coefficient and the significance level was set to p < 0.05. Regression was performed according to the Passing-Bablok method, and confidence intervals calculated with the non-parametric approach given in the original reference.

### Ethics declarations

The study was approved by the local Ethics Committee (Nr. 02/2023) and all participants gave written informed consent. All clinical and biological assessments were carried out in accordance with the Declaration of Helsinki.

## Results

A total of forty-two patients was included in the study. Among these, 27 had AD (with 8 having early onset AD), 1 dementia with Lewy bodies, 3 frontotemporal dementia, 10 other types of dementia, and 1 conversion disorder. Table 1 describes the characteristics of the study population. We could not measure biomarkers in treated saliva because the samples were too dense.

**Table 1:** Characteristics of the study population.

Variable	
Nr	42
Sex, M/F	21/21
Age, yrs	67.50 [10.75]
Disease, nr	
Alzheimer’s disease	27
Conversion disorder	1
Other dementia	14
Biomarkers levels, median [IQR]	
CSF	
Aβ40, pg/mL	8620 [3266.00]
Aβ42, pg/mL	444.0 [267.20]
Aβ42/40 ratio	0.049 [0.03]
pTau, pg/mL	76.00 [89.80]
pTau/Aβ42	0.20 [0.28]
Plasma	
Aβ40, pg/mL	270.9 [44.30]
Aβ42, pg/mL	26.05 [7.25]
Aβ42/40 ratio	0.098 [0.01]
pTau, pg/mL	1.79 [1.25]
pTau/Aβ42	0.07 [0.05]
Saliva	
Aβ40, pg/mL	0.70 [1.19]
Aβ42, pg/mL	0.61 [1.37]
Aβ42/40 ratio	0.775 [0.68]
pTau, pg/mL	5.58 [6.66]
pTau/Aβ42	6.30 [14.94]

**Figure 1:**
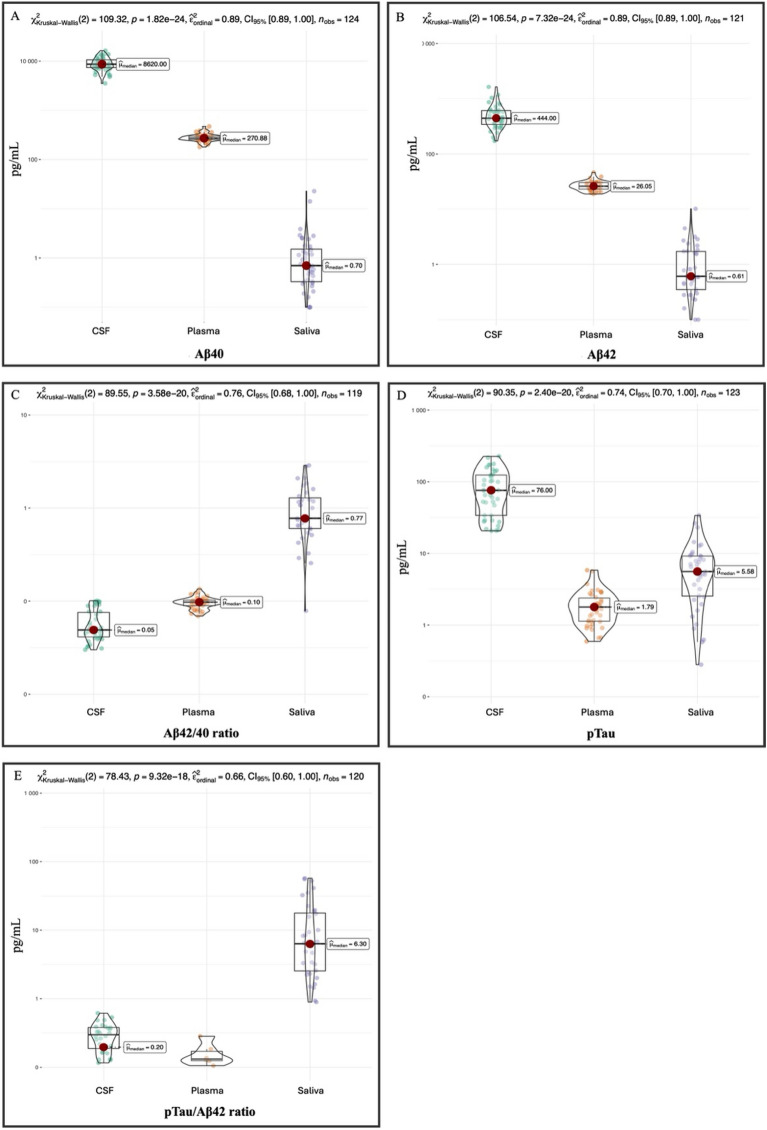
Distribution of Aβ42 (**A**), Aβ40 (**B**), Aβ42/40 (**C**) ratio, pTau (**D**), and pTau/Aβ42 ratio (**E**) levels across different biological matrices, i.e., CSF, plasma, and saliva. Significant differences in median levels of all biomarkers among the three matrices were found. P-value of Kruskal Wallis test is reported above. The effect size was large (epsilon squared, reported above). Holm-adjusted p-values of pairwise comparison among all couple of methods was significant for all pairs.

Figure 1 shows the distribution of Aβ42, Aβ40, Aβ42/40 ratio, pTau, and pTau/Aβ42 ratio levels across the three biological matrices, i.e. CSF, plasma, and saliva. Significant differences in median levels of all biomarkers among the three matrices were found, suggesting a large effect size (p < 0.001).

**Figure 2:**
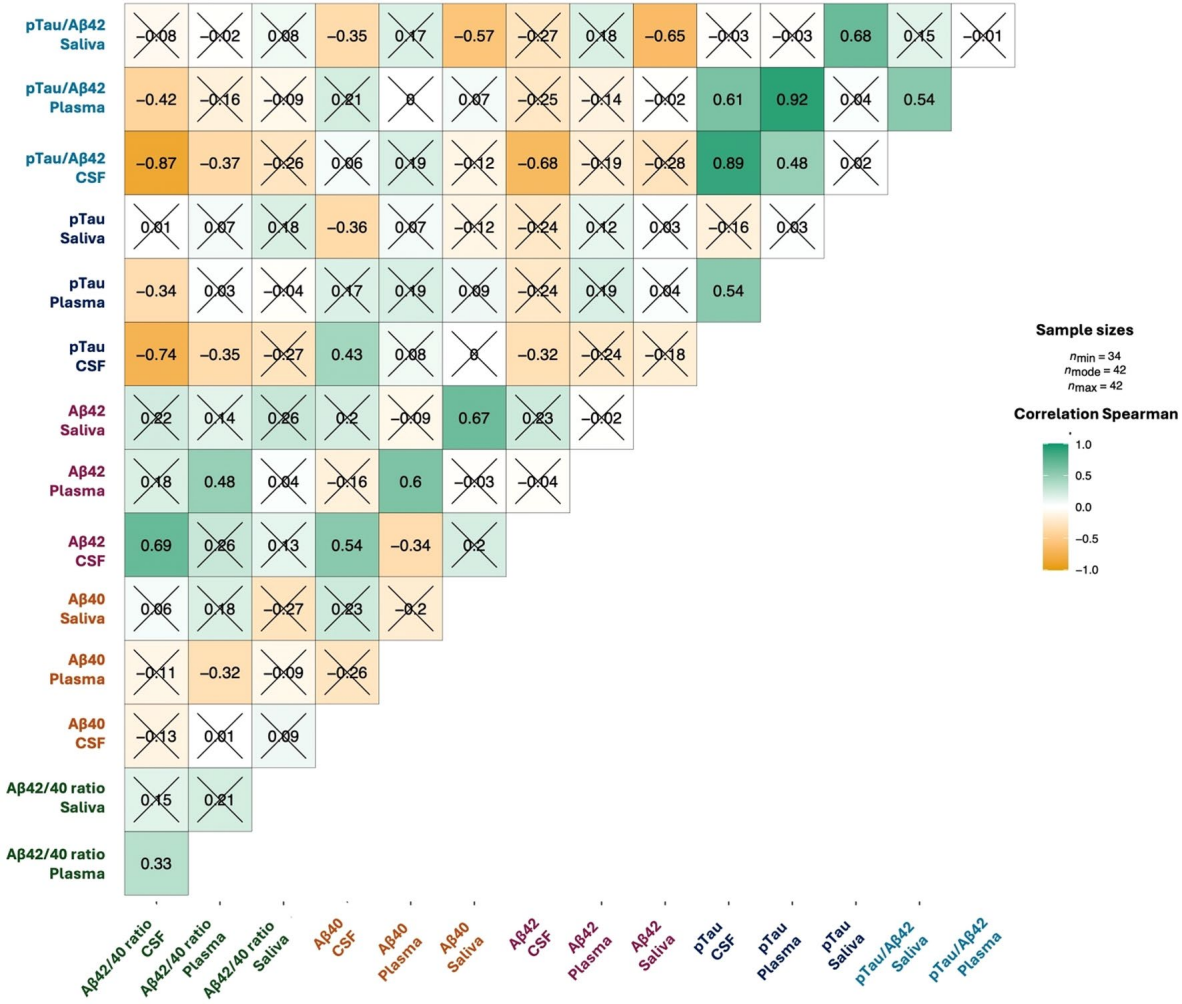
Correlation between biomarkers in the different biological matrices. Non-significant correlations with p-value > 0.05 are crossed. CSF, cerebrospinal fluid; pTau, phosphorylated tau.

The relationship between the different biomarkers across various biofluids was assessed by Spearman correlation (Fig. 2). A positive correlation between pTau levels in CSF and plasma (rho = 0.54 [0.28–0.73]), and Aβ42/40 ratio levels in CSF and plasma (rho = 0.33 [0.02–0.58]) was found. The analysis also indicated significant correlations, both positive and negative, between different analytes in the same matrix (e.g., pTau in CSF vs Aβ42/40 ratio in CSF) or between different analytes in different matrixes (e.g.: pTau in plasma vs Aβ42/40 ratio in CSF), which are represented in Fig. 2 but are beyond the purpose of the current study. The analysis also highlights non-significant correlations, indicating areas where the relationship between biomarkers does not reach statistical significance (crossed, in the figure). Notably, no significant correlations were found between saliva and CSF or saliva and plasma, for all analytes considered.

**Figure 3:**
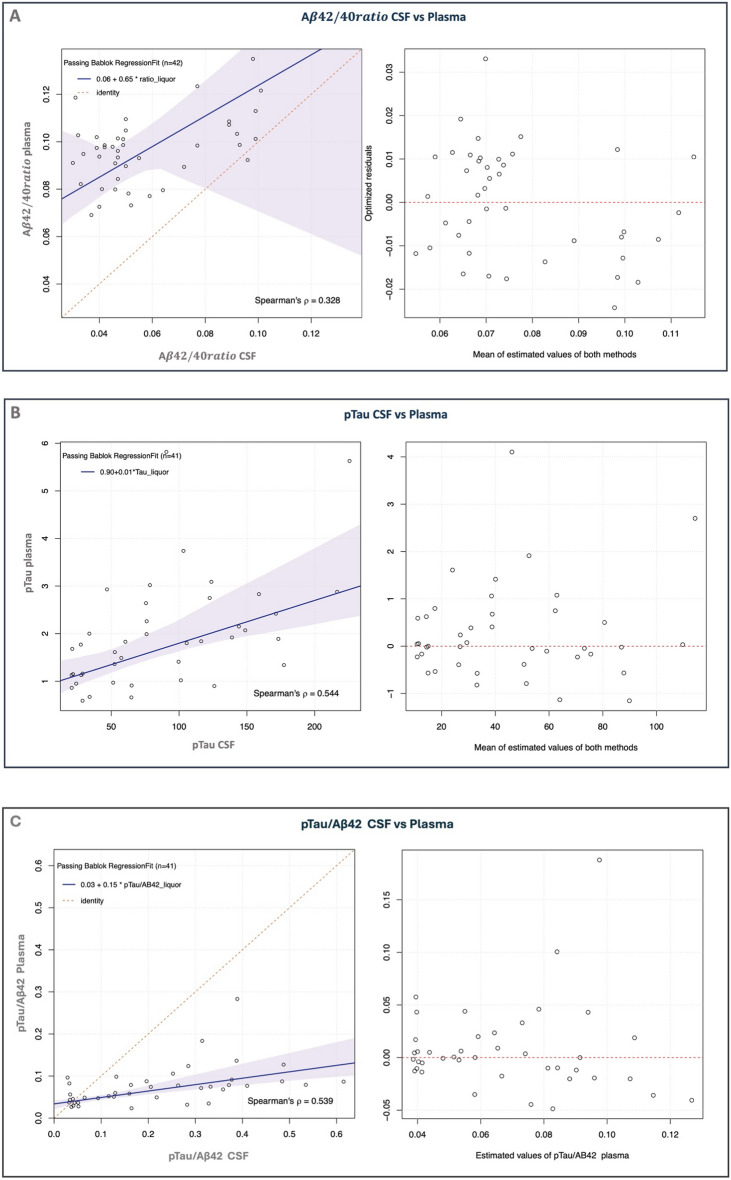
Regression analysis to compare Aβ42/40 ratio and pTau levels in CSF and plasma. In shaded blue the confidence intervals of the regression line. In dashed red the identity line. CSF, cerebrospinal fluid; pTau, phosphorylated Tau.

The correlation analysis only assesses the *strength* of association between variables. To assess the (linear) *nature* of the relationship a regression analysis (Passing-Bablok) was performed for the significant correlations, namely Aβ42/40 ratio in CSF vs plasma, pTau in CSF vs plasma, and pTau/Aβ42 ratio in CSF vs plasma. The results of the regression are shown in Fig. 3.

In the case of Aβ42/40 ratio, the regression equation derived is Aβ42/40 ratio plasma = 0.06 + 0.65 * Aβ42/40 ratio CSF, indicating a significant positive constant bias of 0.06 [0.04–0.07], while the slope is not significant, with a value of 0.65 [− 0.4 to 1.05], encompassing 1 in its large confidence interval. The residual plot does not indicate any major violations of the assumptions necessary for a linear model to be appropriate. However, there seems to be a slight concentration of residuals below the zero line as the mean of estimated values increases, suggesting a potential slight negative bias of the model, which may need further exploration with a larger dataset.

In the case of pTau, the regression equation is pTau plasma = 0.90 + 0.01 * pTau. There is a significant positive constant bias of 0.90 [0.61–1.33] and a slope of 0.01 [0.005–0.014], indicating an increase of plasma pTau of 0.01 per unit of pTau liquor. Such a small slope reflects the huge difference in concentration in the CSF and plasma, with CSF showing a concentration 100-times higher than plasma.

Similarly, the regression equation for the pTau/Aβ42 ratio is pTau/Aβ42 ratio plasma = 0.03 + 0.15 * pTau/Aβ42 ratio CSF. This equation indicates a significant positive constant bias of 0.03 [0.02–0.04] and a slope of 0.15 [0.10–0.26], suggesting that the plasma pTau/Aβ42 ratio increases by 0.15 units for each unit increase in the CSF pTau/Aβ42 ratio. The significant difference in concentration levels between CSF and plasma for these biomarkers is again reflected in the small slope value. The residual plot does not indicate any significant violations of the assumptions.

## Discussion

In this study, we compared CSF, plasma, and saliva levels of AD *core* biomarkers, i.e. Aβ42, Aβ42/40 ratio, pTau, and pTau/Aβ42 ratio measured by Lumipulse G platform, in patients with cognitive decline. The levels of all biomarkers significantly differed in the three biofluids, with saliva showing the lowest and CSF the highest levels of Aβ42, Aβ40, and pTau. In CSF, the median concentration of Aβ40 was 8620 pg/mL, which is approximately 30 times greater than its levels in plasma (270.9 pg/mL). When compared to saliva, where Aβ40 was only 0.70 pg/mL, the concentration in CSF was about 12,000 times higher. The median concentration of Aβ42 in CSF was 444.0 pg/mL, much higher than in plasma (26.05 pg/mL) and saliva (0.61 pg/mL). The Aβ42 CSF level was roughly 20 times that in plasma and about 700 times greater than in saliva. The Aβ42/40 ratio showed significant variation across the biofluids. In CSF, it was relatively low (0.049), indicating a much higher concentration of Aβ40 compared to Aβ42. In plasma, the ratio doubled (0.098), suggesting a slightly different balance between Aβ42 and Aβ40. Saliva, however, showed a drastically higher ratio (0.775), reflecting a much different balance between the two biomarkers in this fluid, albeit at much lower concentrations overall. The median concentration of pTau in CSF was 76.00 pg/mL, significantly higher than in plasma (1.79 pg/mL) and saliva (5.58 pg/mL). The concentration in CSF was about 40 times that in plasma and roughly 15 times that in saliva. Differently from Aβ40 and Aβ42, the pTau appeared to be more concentrated in saliva than in plasma. Interestingly, a positive correlation of pTau and Aβ42/40 ratio levels in CSF and plasma was detected, while no correlation between any biomarker in CSF and saliva was found. Thus, our findings suggest that plasma but not saliva could represent a surrogate biofluid for measuring AD *core* biomarkers. Specifically, plasma Aβ42/40 ratio and pTau could serve as surrogates of the corresponding CSF biomarkers. This is in accordance with Arranz et al. and Martinez-Dubarbie et al., who also explored the correlation between plasma and CSF Aβ42, Aβ42/40 ratio, and pTau levels measured by Lumipulse G platform, finding a moderate correlation for pTau and Aβ42/40 ratio^19,20^.

To date, only Marksteiner et al. measured AD *core* biomarkers in saliva using Lumipulse^21^. Noteworthy, the Authors collected saliva by Salivettes® and they did not detect Aβ42 and Aβ40 due to their binding to cotton. Additionally, they do not state the kit used, i.e. Lumipulse G β-Amyloid 1–40 plasma or CSF, which is important information to understand the sensitivity of the method. Since Fujirebio did not develop a kit for measuring tTau in alternative biological matrices, it is plausible that Authors used kits for CSF. Thus, we cannot compare our findings with those of Marksteiner et al.

Despite the initial enthusiasm for AD biomarkers in saliva, to date evidence in literature are inconsistent to support the use of saliva as a reliable alternative biological matrix to measure AD *core* biomarkers. First, several technical issues related to sample collection and processing limit saliva for diagnostic purposes. Specifically, saliva can be collected from specific salivary glands, such as parotid, or sampling the whole saliva secreted from all the glands. The latter represents the most common and less invasive procedure. In both cases, the samples have the same chemical composition, although the concentration of analytes can vary from one gland to another. Then, it must be established whether to collect unstimulated or stimulated saliva. The choice of the device is another critical issue. The sampling of unstimulated saliva is often preferred because it minimizes the dilution of analytes. Several techniques to collect saliva, including passive drooling and draining, spitting, and swab-based devices, such as Salivette® (blue cap, Sarstedt), which are the most widely used, are available. However, devices could impair the biomarkers analysis. Indeed, it has been documented that Aβ42 and Aβ40 interact with the cotton of the Salivette, making this device unsuitable for AD biomarkers analysis. Once sampled, an open question regards the treatment of saliva with chemical agents to preserve its properties and the related biomarkers. The immediate centrifugation and storage, preferably at − 80 °C, are widely consolidated. In this study, we tried to overcome the known limitations by collecting whole saliva by splitting it into a polypropylene tube, avoiding using devices. We tested both treated and untreated saliva, concluding that the best choice is not to treat saliva.

When using salivary biomarkers, some other considerations should be taken into account. The secretion and composition of saliva may be affected by several factors, including medications, lifestyle, age, sex, and diseases^22,23^. Noteworthy, a bidirectional oral-brain axis connected through almost six routes has been described^22^. Salivary Aβ42 levels may originate from different sources, including cranial nerves innervating salivary glands, acinar epithelial cells of salivary glands, the transportation from blood to saliva, and the presence of Aβ1–42 protein deposits in peripheral regions, such as the nasal mucosa, lacrimal and lingual glands, which could be released directly or indirectly in saliva^24,25^. Two recent meta-analyses showed that the salivary pattern of AD is characterized by elevated Aβ42 levels and unchanged or decreased pTau and tTau levels as compared to controls^25,26^.

Overall, our findings suggest that plasma but not saliva could represent a surrogate biofluid for measuring AD *core* biomarkers. Blood-based AD diagnosis offers several advantages in terms of accessibility and repeatability and the possibility to measure biomarkers by a fully automated platform promotes their widespread diffusion in clinical practice, paving the way to a new revolution in the field of neurodegenerative diseases. Indeed, blood-based biomarkers could aid in supporting Clinicians across the whole path of care of AD patients, from screening, early diagnosis, and monitoring of both disease and therapy. In order to introduce and appropriately use blood-based AD biomarkers in clinical practice, further studies are mandatory to establish reference intervals and decisional cut-offs as well as to evaluate the biological determinants, such as age or sex, and the potential influence of comorbidities. So far, preanalytical variables and their effects on plasma AD *core* biomarkers measured by the Lumipulse platform have been investigated. Musso et al. reported that hemolysis may alter biomarkers levels^27^. They also described the effect of temperature storage (4 °C, − 20 °C, and − 80 °C) on biomarkers concentrations, suggesting that different cut-offs should be used for fresh and thawed samples^27^. However, such an effect was not reported by Mansilla et al.^28^. Thus, further studies are mandatory to clarify the possible effect of temperature storage on plasma biomarkers stability. Two independent studies explored the influence of blood–brain barrier (BBB) integrity, assessed by the CSF/serum albumin quotient, and kidney function on blood-based biomarkers measured by the Lumipulse platform^29,30^. Both found that plasma biomarkers levels are influenced by kidney function, with individuals suffering from renal dysfunction having increased levels. However, the Aβ42/40 ratio spared this effect. This finding is in accordance with Martinez-Dubarbie et al.^20^. On the other hand, they achieved opposite conclusions on the influence of BBB permeability on the biomarkers’ levels^29,30^.

Despite the promising findings, this study has some limitations that should be acknowledged. First, the relatively small sample size limits the generalizability of our findings. Future studies with larger sample sizes are essential to validate our results and enhance the reliability of our conclusions. A significant limitation of our study is the absence of a negative control group comprising individuals without any pathology or with non-neurodegenerative conditions. Due to the invasiveness and the potential risks related to CSF sampling, it is challenging to collect CSF in healthy volunteers or patients with non-neurodegenerative diseases. Thus, we included patients who needed to undergo CSF examinations. Since CSF is the gold standard for evaluating AD biomarkers and testing the reliability of the alternative biological matrices, i.e., plasma and saliva, we did not include negative controls, which could provide only plasma and saliva but not CSF samples.

In conclusion, the measurement of AD *core* biomarkers in plasma by fully automated platform hold great promise for routine clinical use^31^. However, some issues must be resolved before their introduction in clinical laboratories.

## Data Availability

The datasets generated and analysed during the current study are not publicly available due to restrictions from our Institution but are available from the corresponding author on reasonable request.

## References

[1] GBD 2019 Dementia Forecasting Collaborators (2022). Estimation of the global prevalence of dementia in 2019 and forecasted prevalence in 2050: An analysis for the Global Burden of Disease Study 2019. Lancet Public health..

[2] McKeown A (2020). Health outcome prioritization in Alzheimer's disease: Understanding the ethical landscape. J. Alzheimer's Dis..

[3] Mahaman YAR (2022). Biomarkers used in Alzheimer's disease diagnosis, treatment, and prevention. Ageing Res. Rev..

[4] Piccoli T (2022). Biomarkers related to synaptic dysfunction to discriminate Alzheimer's disease from other neurological disorders. Int. J. Mol. Sci..

[5] Jack CR (2018). NIA-AA research framework: Toward a biological definition of Alzheimer's disease. Alzheimers Dementia..

[6] Hansson O, Lehmann S, Otto M, Zetterberg H, Lewczuk P (2019). Advantages and disadvantages of the use of the CSF amyloid β (Aβ) 42/40 ratio in the diagnosis of Alzheimer's disease. Alzheimer's Res. Ther..

[7] Wachholz P, Skowronek R, Pawlas N (2023). Assessing the applicability of cerebrospinal fluid collected from the spinal cord for the determination of ethyl alcohol in post-mortem toxicology. Forensic Sci. Med. Pathol..

[8] Agnello L (2022). Evaluation of alpha-synuclein cerebrospinal fluid levels in several neurological disorders. J. Clin. Med..

[9] Agnello L (2021). Neurogranin as a reliable biomarker for synaptic dysfunction in Alzheimer's disease. Diagnostics..

[10] Pais MV, Forlenza OV, Diniz BS (2023). Plasma biomarkers of Alzheimer's disease: A review of available assays, recent developments, and implications for clinical practice. J. Alzheimer's Dis. Rep..

[11] Janelidze S (2023). Head-to-head comparison of 10 plasma phospho-tau assays in prodromal Alzheimer's disease. Brain..

[12] Nijakowski K, Owecki W, Jankowski J, Surdacka A (2024). Salivary biomarkers for Alzheimer's disease: A systematic review with meta-analysis. Int. J. Mol. Sci..

[13] Bermejo-Pareja F, Antequera D, Vargas T, Molina JA, Carro E (2010). Saliva levels of Abeta1-42 as potential biomarker of Alzheimer's disease: A pilot study. BMC Neurol..

[14] Shi M (2011). Salivary tau species are potential biomarkers of Alzheimer's disease. J. Alzheimer's Dis..

[15] Wolgin M, Zobernig M, Dvornyk V, Braun RJ, Kielbassa AM (2022). Systematic review on saliva biomarkers in patients diagnosed with morbus Alzheimer and morbus Parkinson. Biomedicines..

[16] McKhann GM (2011). The diagnosis of dementia due to Alzheimer's disease: Recommendations from the National Institute on Aging-Alzheimer's Association workgroups on diagnostic guidelines for Alzheimer's disease. Alzheimers Dementia..

[17] Albert MS (2011). The diagnosis of mild cognitive impairment due to Alzheimer's disease: Recommendations from the National Institute on Aging-Alzheimer's Association workgroups on diagnostic guidelines for Alzheimer's disease. Alzheimers Dementia..

[18] Hansson O (2021). The Alzheimer's Association international guidelines for handling of cerebrospinal fluid for routine clinical measurements of amyloid β and tau. Alzheimers Dementia..

[19] Arranz J (2023). Diagnostic performance of plasma pTau 217, pTau 181, Aβ 1–42 and Aβ 1–40 in the LUMIPULSE automated platform for the detection of Alzheimer disease. Res. Sq..

[20] Martínez-Dubarbie F, Guerra-Ruiz A, López-García S, Irure-Ventura J, Lage C, Fernández-Matarrubia M (2024). Influence of physiological variables and comorbidities on plasma Aβ40, Aβ42, and p-tau181 levels in cognitively unimpaired individuals. Int. J. Mol. Sci..

[21] Marksteiner J, Defrancesco M, Humpel C (2022). Saliva tau and phospho-tau-181 measured by Lumipulse in patients with Alzheimer's disease. Front. Aging Neurosci..

[22] Zürcher C, Humpel C (2023). Saliva: A challenging human fluid to diagnose brain disorders with a focus on Alzheimer's disease. Neural Regener. Res..

[23] Takeda I (2009). Understanding the human salivary metabolome. NMR Biomedicine..

[24] Oh YS, Turner RJ (2006). Turner, Effect of gamma-secretase inhibitors on muscarinic receptor-mediated calcium signaling in human salivary epithelial cells. Am. J. Physiol..

[25] Kaufman E, Lamster IB (2002). The diagnostic applications of saliva—a review. Crit. Rev. Oral Biol. Med..

[26] Fan Z (2023). Salivary Aβ1-42 may be a quick-tested biomarker for clinical use in Alzheimer's disease: A meta-analysis. J. Neurol..

[27] Musso G, Cosma C, Zaninotto M, Gabelli C, Basso D, Plebani M (2022). Pre-analytical variability of the Lumipulse immunoassay for plasma biomarkers of Alzheimer's disease. Clin. Chem. Lab. Med..

[28] Mansilla A (2023). Effects of storage conditions on the stability of blood-based markers for the diagnosis of Alzheimer's disease. Clin. Chem. Lab. Med..

[29] Verde F (2023). Influence of kidney function and CSF/serum albumin ratio on plasma Aβ42 and Aβ40 levels measured on a fully automated platform in patients with Alzheimer's disease. Neurol. Sci..

[30] Bellomo G (2024). Fully automated measurement of plasma Aβ42/40 and p-tau181: Analytical robustness and concordance with cerebrospinal fluid profile along the Alzheimer's disease continuum in two independent cohorts. Alzheimers Dementia..

[31] Agnello L (2020). Diagnostic accuracy of cerebrospinal fluid biomarkers measured by chemiluminescent enzyme immunoassay for Alzheimer disease diagnosis. Scand. J. Clin. Lab. Investig..

